# Melanoma and other tumors of the skin among office, other indoor and outdoor workers in Sweden 1961-1979.

**DOI:** 10.1038/bjc.1986.80

**Published:** 1986-04

**Authors:** D. Vågero, G. Ringbäck, H. Kiviranta

## Abstract

Through a record linkage of the 1960 Swedish Population Census and the 1961-79 Cancer Registry it was possible to analyse the occurrence of melanoma and other skin tumours by occupations, classified as either outdoor, office or other indoor work. Office work as compared to other indoor work was associated with risk of melanoma of the covered, but not the uncovered, parts of the body. It is shown that social class is a confounding factor in such analysis, but the elevated risk of melanoma of covered parts of the body among office workers is not entirely due to their higher social class.


*Br. J. Cancer* (1986), **53**, 507–512

### Melanoma and other tumours of the skin among office, other indoor and outdoor workers in Sweden 1961–1979

D. Vågerö[1,3], G. Ringbäck[1] & H. Kiviranta[2]

[1]*Dept. of Social Medicine, Karolinska Institute/Huddinge University Hospital, Stockholm, Sweden;* [2]*Dept. of Epidemiology, National Institute of Environmental Medicine, Stockholm, Sweden;* [3]*Dept. of Epidemiology, London School of Hygiene and Tropical Medicine, London, UK.*

**Summary** Through a record linkage of the 1960 Swedish Population Census and the 1961–79 Cancer Registry it was possible to analyse the occurrence of melanoma and other skin tumours by occupations, classified as either outdoor, office or other indoor work. Office work as compared to other indoor work was associated with risk of melanoma of the covered, but not the uncovered, parts of the body. It is shown that social class is a confounding factor in such analysis, but the elevated risk of melanoma of covered parts of the body among office workers is not entirely due to their higher social class.

Malignant melanoma of the skin is the tumour whose incidence is increasing most rapidly in Sweden (National Board of Helth and Welfare, 1980*b*). This is suggested as being the result of changes in fashion and exposure to sunshine (Magnus, 1977).

The relation of sun exposure to risk for malignant melanoma of the skin is, however, not straightforward and there has been conflicting evidence (Anonymous, 1981; Lee, 1982). Some recent and large-scale case-control studies demonstrated a higher risk for those with intermittent episodes of sunburn (MacKie & Aitchinson, 1982; Elwood *et al.*, 1984; Elwood *et al.*, 1985). Unlike for other skin cancers, cumulative doses of sun exposure are now often thought to be of little or no importance because, for instance, some indoor occupations have very high risks while outdoor occupations have not (Mackie, 1983). However, there are two recent studies, both from Australia, suggesting that there is indeed a higher risk for those having a large lifetime dose of sun exposure (Green, 1984; Holman & Armstrong, 1984). Elwood *et al.* (1985) on the other hand suggest that long term constant exposure has no effect or may be protective. This suggestion was based on analysis of sun exposure patterns in Western Canada. If the ultraviolet radiation (UV-B) penetrating the epidermis is a causal agent it seems reasonable that a high number of severe sunburns and a very large lifetime dose could both independently be indicators of risk.

The pattern of occurrence has revealed other environmental factors strongly linked to the onset of melanoma. It is more common in upper than in lower social classes (Logan, 1982; Lee & Strickland, 1980; Vågerö & Persson, 1984). It is not known why this is the case but it is usually assumed that this is due to different patterns of sun exposure and holiday travel. Several studies have discussed the relationship of melanoma to outdoor and indoor work (Lee & Strickland, 1980; Klepp & Magnus, 1979; Beral & Robinson, 1981; Cooke *et al.*, 1984). Klepp & Magnus (1979) for instance hypothesised that outdoor work would indicate risk because it meant more exposure to the sun. They also found support for that hypothesis since there were more outdoor workers among cases when compared to controls. A recent study from New Zealand (Cooke *et al.*, 1984) found no difference between outdoor and indoor occupations other than that resulting from social class.

The most comprehensive study undertaken so far, dealing with the differences between indoor and outdoor workers was based on cases in England and Wales. This demonstrated that office workers had the highest risk of malignant melanoma of the skin and that outdoor workers had the lowest risk (Beral & Robinson, 1981). Moreover, this was largely attributable to a difference in risk for melanoma of covered parts of the body. The same study also gave evidence that the difference in that risk between groups of indoor and outdoor workers was present within the same social class. This would then indicate that independent risk factors operate and are reflected in the above differences. For instance, it may be suggested that the cumulative effects of undiffused fluorescent lighting, prevalent among a number of indoor occupations, could be such a risk factor (Beral *et al.*, 1982; Williamson & Elwood, 1984). Alternatively, within each social class, a higher proportion of indoor workers may get sunburnt on their annual holiday than the corresponding proportion among outdoor workers.

Correspondence: D. Vågerö.
Received 27 August 1985; & in revised form, 5 December 1985.

© The Macmillan Press Ltd., 1986

The purpose of the present study was to compare office, other indoor, and outdoor workers in Sweden with respect to melanoma risk. In addition, other skin tumours were analysed for comparison.

We wanted to find out if the results based on data from England and Wales (Beral & Robinson, 1981) could be reproduced from Swedish data, by taking advantage of a nation-wide population-based registry, containing cases diagnosed and registered between 1961 and 1979.

### Population under study and methods

The analysis was based on cases diagnosed as malignant melanoma in Sweden between 1961–1979. In addition, cases of basal cell and squamous cell carcinoma for the same period were analysed. The cancer cases were obtained from the extended Swedish Cancer Environment Registry, created from a linkage of the Swedish Cancer Registry to the Population Census of 1960 (National Board of Health and Welfare, 1980*a*; CMR-nämnden, 1983). For each case, census information such as occupation and county of residence (in 1960) was known. For each combination of variables in the Census the number of persons was known and thus the population at risk at the beginning of the follow-up period could be established with great accuracy.

The study was restricted to all men and women born between 1896–1940 who were classified as economically active at the Census date. Thus the population under study was 2,630,458 persons in all. These were classified into three main groups by occupation: office workers, other indoor workers and outdoor workers. A small group of occupations could not be classified as any one of these and was excluded from the study. The classification was made prior to analysing the Swedish data set, without knowledge of how occupations had been classified in the previous British study, by someone who was not familiar with its results. In the Census the occupations were coded according to the 'Nordic Classification of Occupations' (Nordisk Yrkesklassificering, 1974). For each such code there is a description of that work. These descriptions were the basis for our classification into the above mentioned three groups. The size of each group is seen in Table I.

Those occupations where a large part of the day was spent in outdoor daylight were classified as outdoor work. A large such group was farmers among men and farm workers among women. Office workers included those who spent most of their day in an office environment. Banks, post offices, schools and libraries were also considered as examples of such an environment. Typical and large groups were engineers/technicians among men and secretaries among women. Other indoor workers were those who spent most of the day indoors in a non-office environment, for example, a shop, a factory, a hospital or a laboratory. Such groups were, for instance, mechanics among men and shop assistants among women.

There were 4,706 cases of malignant melanoma of the skin (ICD-code 190, 7th rev.). These were later divided into melanomas of the uncovered parts of the body (190.1–190.4) and melanomas of the covered parts of the body (190.5–190.7) and analysed separately. Melanomas of unknown or multiple localization (190.8) were excluded in the site specific analysis. There were also 4,244 cases of basal cell and squamous cell cancers, which had entered the Registry as malignant cases (ICD-code 191 and histological codes 126 or 146). These were analysed for comparison.

The observed number of cases for a specific

**Table I** Malignant melanoma of the skin, all sites. Cases 1961–79. Morbidity ratios standardized for age and county of residence

| *Type of work* | *Gender* | *Size of group* | *Number of cases* obs | exp | *SMorbR*[a] | *95% confidence limits* |
|---|---|---|---|---|---|---|
| Office | m | 348,424 | 882 | 637 | 139 | 130–148 |
| | f | 218,969 | 482 | 406 | 119 | 108–130 |
| | m+f | 567,393 | 1,364 | 1,043 | 131 | 124–138 |
| Indoor, non-office | m | 914,693 | 1,484 | 1,572 | 94 | 90–99 |
| | f | 517,858 | 942 | 1,011 | 93 | 87–99 |
| | m+f | 1,432,551 | 2,426 | 2,583 | 94 | 90–98 |
| Outdoor | m | 594,418 | 854 | 992 | 86 | 80–92 |
| | f | 36,096 | 62 | 72 | 86 | 66–110 |
| | m+f | 630,514 | 916 | 1,065 | 86 | 81–92 |

[a]Standardized morbidity ratio.

diagnosis was compared to the expected number and the standardized morbidity ratio (SMorbR) calculated. A 95% confidence interval was calculated by the method suggested by Rothman and Boice (1982). The expected numbers were based on rates for the entire working population of the same sex, age (5-year age groups) and residence (county of residence) and the total expected value was obtained by summarizing over strata. Thus the SMorbRs presented are always standardized for age and residence.

In a further analysis, social class as defined from the Census information (Vågerö & Persson, 1984) was adjusted for.

### Results

When analysed as one category, malignant melanoma of the skin is clearly more frequent among office workers than in any other group. The outdoor group SMorbR is particularly low (Table I).

Looking at the covered parts of the body, the contrast between the high SMorbR of office workers and the low SMorbR of other indoor and of outdoor workers is even more striking (Table II). For the uncovered parts of the body, these contrasts do not exist – if anything, there may be a moderately elevated risk for outdoor workers (Table III). For non-melanoma skin cancers there may be a moderately raised risk among outdoor workers as well as among office workers, while other indoor workers seem to be at somewhat less risk (Table IV).

Social classes in Sweden are known to have quite different risks for the onset of malignant melanoma. Table V is based on a representative sample of the Swedish population interviewed for the Stockholm Institute of Social Research longitudinal study (Johansson, 1973). It can be seen

**Table II** Malignant melanoma on covered parts of the body. Cases 1961–79. Morbidity ratios standardized for age and county of residence

| *Type of work* | *Gender* | *Number of cases* | | *SMorbR*[a] | *95% confidence limits* |
|---|---|---|---|---|---|
| | | *Obs* | *Exp* | | |
| Office | m | 677 | 473 | 143 | 133–154 |
| | f | 385 | 321 | 120 | 108–133 |
| | m+f | 1,062 | 794 | 134 | 126–142 |
| Indoor, non-office | m | 1,096 | 1,162 | 94 | 89–100 |
| | f | 725 | 785 | 92 | 86–99 |
| | m+f | 1,821 | 1,947 | 94 | 89–98 |
| Outdoor | m | 575 | 709 | 81 | 75–88 |
| | f | 45 | 55 | 82 | 60–109 |
| | m+f | 620 | 764 | 81 | 75–88 |

[a]Standardized morbidity ratio.

**Table III** Malignant melanoma on uncovered parts of the body. Cases 1961–79. Morbidity ratios standardized for age and county of residence

| *Type of work* | *Gender* | *Number of cases* | | *SMorbR*[a] | *95% confidence limits* |
|---|---|---|---|---|---|
| | | *Obs* | *Exp* | | |
| Office | m | 89 | 89 | 100 | 80–123 |
| | f | 53 | 50 | 106 | 79–139 |
| | m+f | 142 | 139 | 102 | 86–120 |
| Indoor, non-office | m | 215 | 222 | 97 | 84–111 |
| | f | 137 | 140 | 98 | 82–116 |
| | m+f | 352 | 361 | 98 | 88–108 |
| Outdoor | m | 175 | 162 | 108 | 93–125 |
| | f | 11 | 12 | 92 | 46–164 |
| | m+f | 186 | 174 | 107 | 92–123 |

[a]Standardized morbidity ratio.

**Table IV** Squamous and basal cell carcinomas. Cases 1961–79. Morbidity ratios standardized for age and county of residence

| Type of work | Gender | Number of cases Obs | Exp | SMorbR[a] | 95% confidence limits |
|---|---|---|---|---|---|
| Office | m | 713 | 638 | 112 | 104–120 |
| | f | 177 | 148 | 119 | 103–138 |
| | m+f | 890 | 786 | 113 | 106–121 |
| Indoor, non-office | m | 1,440 | 1,558 | 91 | 86–96 |
| | f | 435 | 454 | 96 | 87–105 |
| | m+f | 1,875 | 2,042 | 92 | 88–96 |
| Outdoor | m | 1,442 | 1,364 | 106 | 100–111 |
| | f | 37 | 42 | 89 | 62–122 |
| | m+f | 1,479 | 1,406 | 105 | 100–111 |

[a]Standardized morbidity ratio.

**Table V** 'Holidaying in Southern Europe last year'. Proportions in three social classes according to interviews at 1968, 1974 and 1981. (Stockholm Institute of Social Research longitudinal study)

| Proportion affirmative | Year of interview 1968 | 1974 | 1981 |
|---|---|---|---|
| Soc cl I (highest) | 13.5% | 22.5% | 16.8% |
| Soc cl II (middle) | 7.9% | 12.6% | 11.4% |
| Soc cl III (lowest) | 3.9% | 7.4% | 9.3% |

**Table VI** 'Have you visited an open air swimming pool or done any other kind of swimming out of doors during the last year?' (Statistics, Sweden)

| | Never | Yes, occasionally | Yes, regularly (>20 times) |
|---|---|---|---|
| Soc cl I (highest) | 39.0% | 36.1% | 24.9% |
| Soc cl II (middle) | 13.0% | 38.6% | 48.2% |
| Soc cl III (lowest) | 28.1% | 35.3% | 36.7% |

that holidaying in southern Europe is persistently a less common feature of life among the lower classes. Other leisure time activities that may involve intermittent exposure to the sun are nowadays most widespread among the middle class. Table VI demonstrates this for a representative sample interviewed by Statistics, Sweden during 1982. Patterns of sun exposure is one, but maybe not the only, underlying explanation for the social distribution of melanoma.

In analysing differences between office, other indoor, and outdoor workers, we were hoping to rule out confounding by social class by adjusting for the social class composition of each of these groups (Table VII). The results are consistent with a somewhat higher risk of melanoma of the face and neck for outdoor workers. For office workers the observed number was lower than expected.

Melanomas of the covered parts of the body showed a significant deficit among outdoor workers. Among indoor workers there was a difference between office and other indoor workers. Office workers had an elevated risk of melanoma for covered parts while this was not true for other indoor workers. Comparing office and other indoor workers directly across all classes gives an estimate of a higher risk of at least 10% in the office group.

The suggested elevated risk for squamous and basal cell cancers among office workers is mainly due to confounding by social class as can be seen in Table VII.

Table VII presents results for men and women combined. Analysing each gender separately did not lead to different results or conclusions, although the difference between office and non-office indoor workers with regard to melanoma of covered parts seemed somewhat more pronounced among men than among women.

### Discussion

The results show that there is a higher than expected incidence of melanoma among office

**Table VII** Morbidity ratios standardized for age, gender, county of residence and social class. Cases 1961–79.

| | | *Malignant melanoma of uncovered parts* | | | |
|---|---|---|---|---|---|
| *Type of work* | *Gender* | *Number of cases Obs* | *Exp* | *SMorbR*[a] | *95% confidence limits* |
| Office | m+f | 142 | 156.0 | 91 | 77–107 |
| Indoor, non-office | m+f | 352 | 347.5 | 101 | 91–112 |
| Outdoor | m+f | 186 | 170.0 | 109 | 94–126 |
| | | *Malignant melanoma of covered parts* | | | |
| *Type of work* | *Gender* | *Number of cases Obs* | *Exp* | *SMorbR*[a] | *95% confidence limits* |
| Office | m+f | 1,062 | 980.3 | 108 | 102–115 |
| Indoor, non-office | m+f | 1,821 | 1,816.2 | 100 | 96–105 |
| Outdoor | m+f | 620 | 690.3 | 90 | 83–97 |
| | | *Squamous and basal cell cancers* | | | |
| *Type of work* | *Gender* | *Number of cases Obs* | *Exp* | *SMorbR*[a] | *95% confidence limits* |
| Office | m+f | 890 | 867.5 | 103 | 96–110 |
| Indoor, non-office | m+f | 1,875 | 1,970.2 | 95 | 91–100 |
| Outdoor | m+f | 1,479 | 1,394.1 | 106 | 101–112 |

[a]Standardized morbidity ratio.

workers, but not for other indoor workers. Outdoor workers have a low incidence. These differences are almost entirely due to a striking contrast in incidence of melanoma on covered parts of the body. For melanomas of uncovered parts of the body, as for basal cell and squamous cell cancers, there was some extra risk among outdoor workers.

Thus these results are similar to those presented earlier by Beral and Robinson (1981), in spite of the fact that the incidence rate in Sweden was in general more than twice that in England and Wales (Lee & Issenberg, 1972; Waterhouse *et al.*, 1976). The seemingly elevated risk for squamous and basal cell cancers among office workers in our study is, however, not in accordance with that earlier study. However, the analysis shows this high risk to be largely due to confounding by social class. It is also possible that different criteria for including non-melanoma skin cancers account for some of the discrepancy. Our results also suggest that the elevated risk of melanoma on covered parts of the body for office workers is not entirely due to their higher social class. We estimate that indoor office workers as compared to other indoor workers may have a 10% or more elevated incidence after taking into account differences in age, residence and social class distribution. It is also clear that comparing groups of indoor and outdoor workers without taking social class into account introduces confounding in the analysis. It has not been possible, on the basis of this study, to disentangle further any independent effects of office work and social class.

Our interpretation is that differences between office, other indoor, and outdoor workers do not merely reflect such general risk differences between social classes as are assumed to be caused by different patterns of sun exposure. In particular, such differences would not explain the contrast between office and non-office indoor workers within the same social class which have now been suggested by three studies (this one; Lee & Strickland, 1980; Beral & Robinson, 1981). However, the possibility that within each social class, patterns of sunlight exposure and experience of sunburn are different in office, other indoor and outdoor workers, could not be entirely ruled out.

It is not likely that there are any genetic or constitutional differences between those groups compared in this study that could explain its result. Sweden is relatively homogenous genetically and there is no reason to believe that the distribution of naevi, pigmentation, or other such risk indicators co-variate with groups of office, other indoor, or outdoor workers.

This study was supported by a research fellowship to one of the authors (DV) from the Swedish Council for Coordination and Planning of Research (FRN). We are grateful to the Stockholm Institute of Social Research and to Statistics, Sweden, for access to their interview data.

### References

ANONYMOUS (1981). The aetiology of melanoma. *Lancet*, **i**, 253.

BERAL, V. & ROBINSON, N. (1981). Relationship of malignant melanoma, basal and squamous skin cancers to indoor and outdoor work. *Br. J. Cancer*, **44**, 886.

BERAL, V., EVANS, S., SHAW, H. & MILTON, G. (1982). Malignant melanoma and exposure to fluorescent lighting at work. *Lancet*, **ii**, 290.

CMR-NÄMNDEN (1983). Cancer-miljöregistret uppdaterat. Omfattar nu över 600,000 fall. *Läkartidningen*, **80**, 123.

COOKE, K.R., SKEGG, D.C.G. & FRASER, J. (1984). Socioeconomic status, indoor and outdoor work and malignant melanoma. *Int. J. Cancer*, **34**, 57.

ELWOOD, J.M., GALLAGHER, R.P., HILL, G.B. & 3 others. (1984). Pigmentation and skin reaction to sun as risk factors for cutaneous malignant melanoma. Western Canada Melanoma Study. *Br. Med. J.*, **288**, 99.

ELWOOD, J.M., GALLAGHER, R.P., HILL, G.B. & PEARSON, J. (1985). Cutaneous melanoma in relation to intermittent and constant sun exposure – the Western Canada Melanoma Study. *Int. J. Cancer*, **35**, 427.

GREEN, A. (1984). Sun exposure and the risk of melanoma. *Aust. J. Derm.*, **25**, 99.

HOLMAN, C.D. & ARMSTRONG, B.K. (1984). Cutaneous malignant melanoma and indicators of total accumulated exposure to the sun. An analysis separating histogenic types. *J. Natl. Cancer. Inst.*, **73**, 75.

JOHANSSON, S. (1973). The level of living survey. A presentation. *Acta Sociologica*, **3**, 211.

KLEPP, O. & MAGNUS, K. (1979). Some environmental and bodily characteristics of melanoma patients. A case-control study. *Int. J. Cancer*, **23**, 482.

LEE, J.A.H. & ISSENBERG, H.J.A. (1972). A comparison between England and Wales and Sweden in the incidence and mortality of malignant skin tumours. *Br. J. Cancer*, **26**, 56.

LEE, J.A.H. & STRICKLAND, D. (1980). Malignant melanoma, social status and outdoor work. *Br. J. Cancer*, **41**, 757.

LEE, J. (1982). Melanoma and exposure to sunlight. *Epidemiologic Reviews*, **4**, 110.

LOGAN, W.P.D. (1982). Cancer mortality by occupation and social class 1851–1971. *IARC Scientific Publications No. 36*. London and Lyon.

MACKIE, R.M. & AITCHINSON, T. (1982). Severe sunburn and subsequent risk of primary cutaneous malignant melanoma in Scotland. *Br. J. Cancer*, **46**, 955.

MACKIE, R.M. (1983). The pathogenesis of cutaneous malignant melanoma. *Br. Med. J.*, **287**, 156.

MAGNUS, K. (1977). Incidence of malignant melanoma of the skin in the five Nordic countries. Significance of solar radiation. *Int. J. Cancer*, **20**, 477.

NATIONAL BOARD OF HEALTH AND WELFARE (1980*a*). The National Central Bureau of Statistics, The Swedish Work Environment Fund. *Swedish Cancer Environment Registry 1961–1973*. SOS: Stockholm.

NATIONAL BOARD OF HEALTH AND WELFARE (1980*b*). *The Cancer Registry. Cancer Incidence in Sweden 1978*. SOS: Stockholm.

NORDISK YRKESKLASSIFICERING (1974). Helsingborg, Sweden.

ROTHMAN, K. & BOICE, I. (1982). Epidemiologic analysis with a programmable calculator. Epidemiology Resources Inc.: Boston.

VÅGERÖ, D. & PERSSON, G. (1984). Risks, survival and trends of malignant melanoma among white and blue collar workers in Sweden. *Soc. Sci. Med.*, **19**, 475.

WATERHOUSE, J.A.H., MUIR, C.S., CORREA, P. & POWELL, J. (1976). Cancer incidence in five continents. Vol. 3. *IARC Scientific Publications No. 16*: Lyon.

WILLIAMSON, C. & ELWOOD, J.M. (1984). Malignant melanoma and exposure to fluorescent and other lighting. Paper read at the Society for Social Medicine annual scientific meeting in Oxford, 19–21 September 1984.


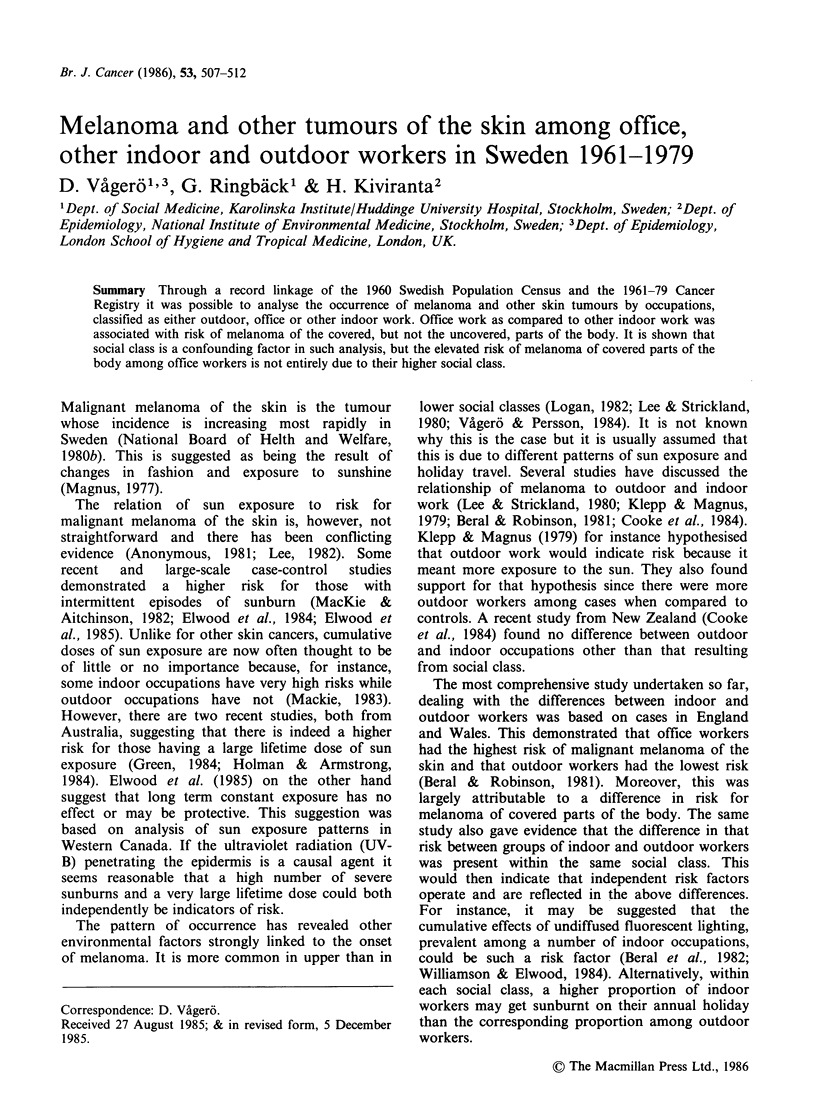

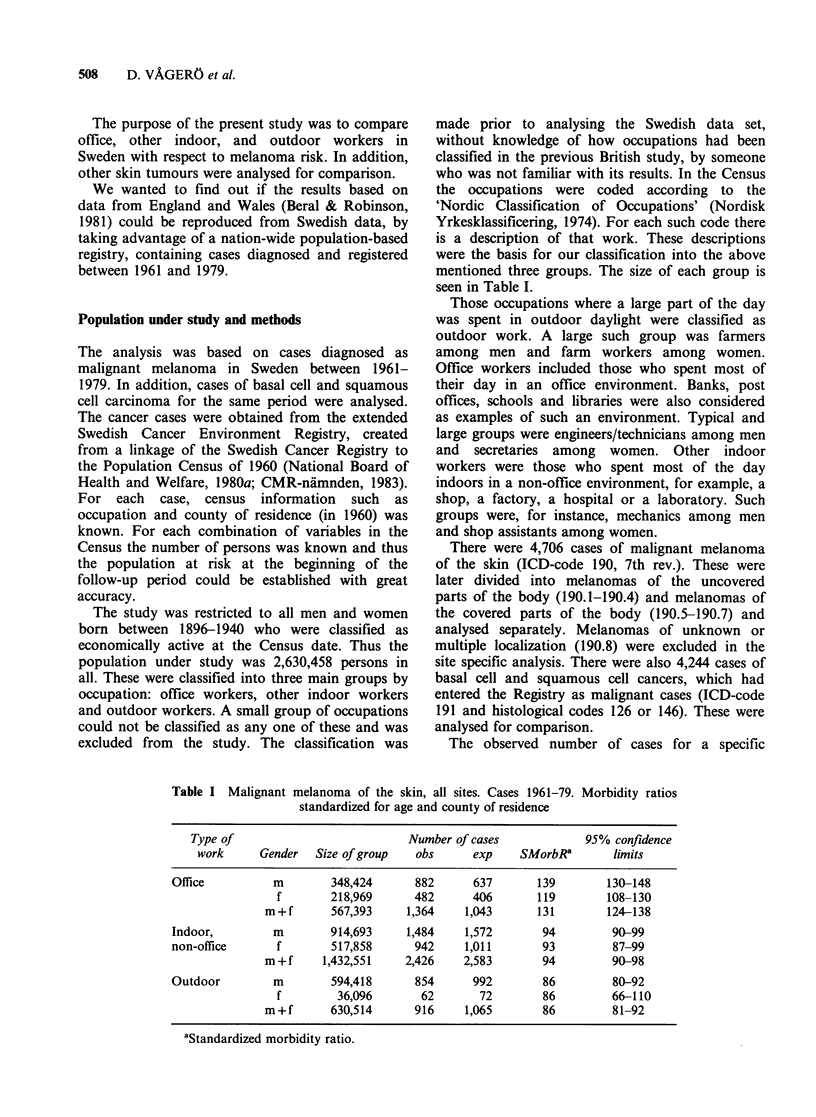

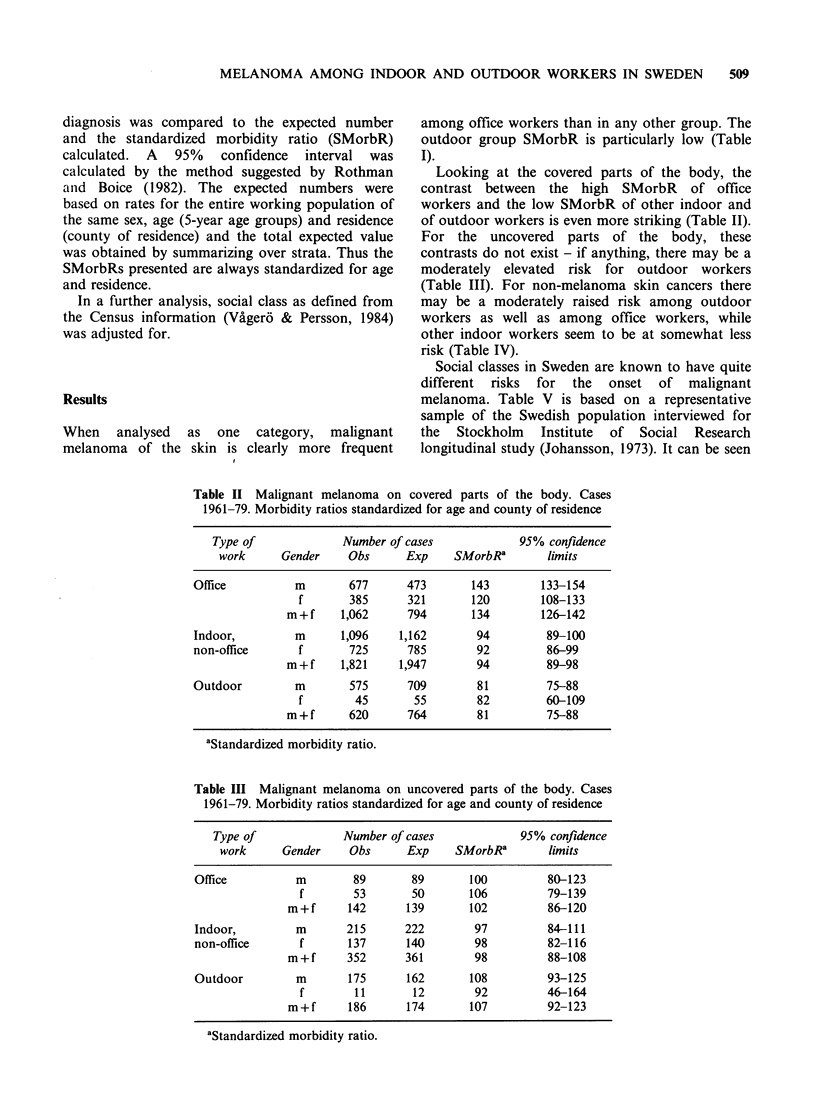

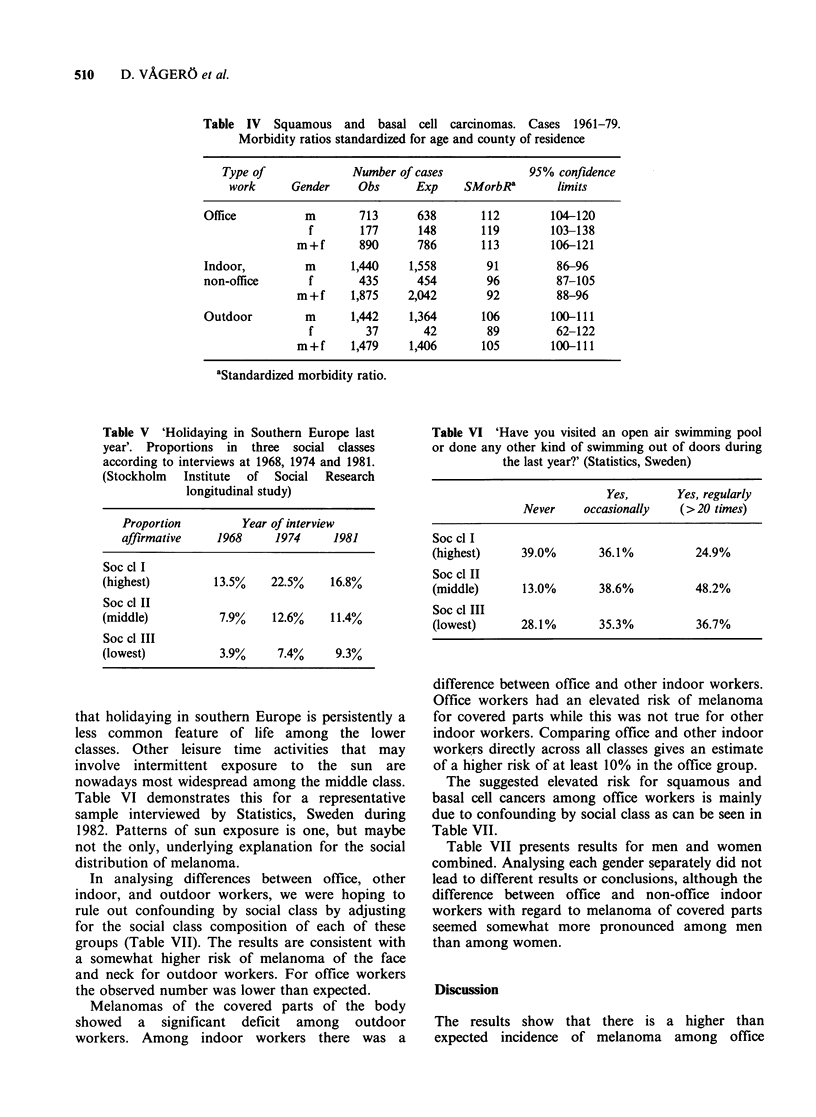

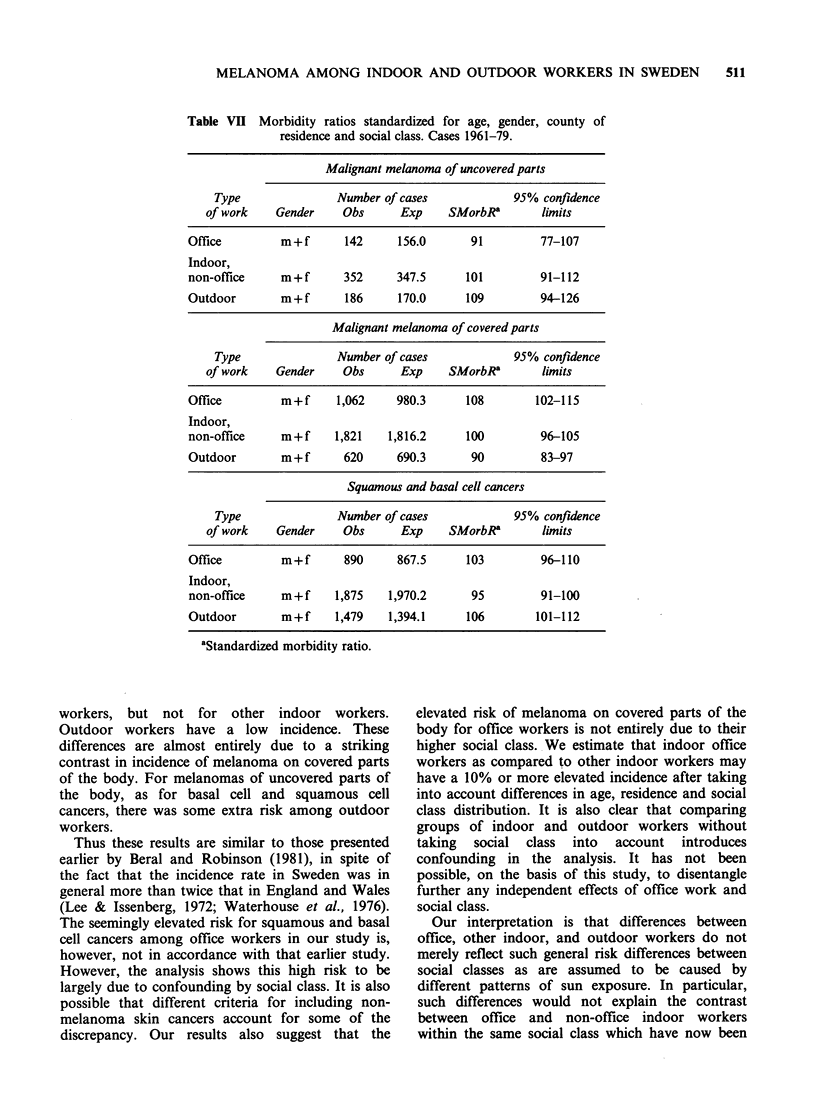

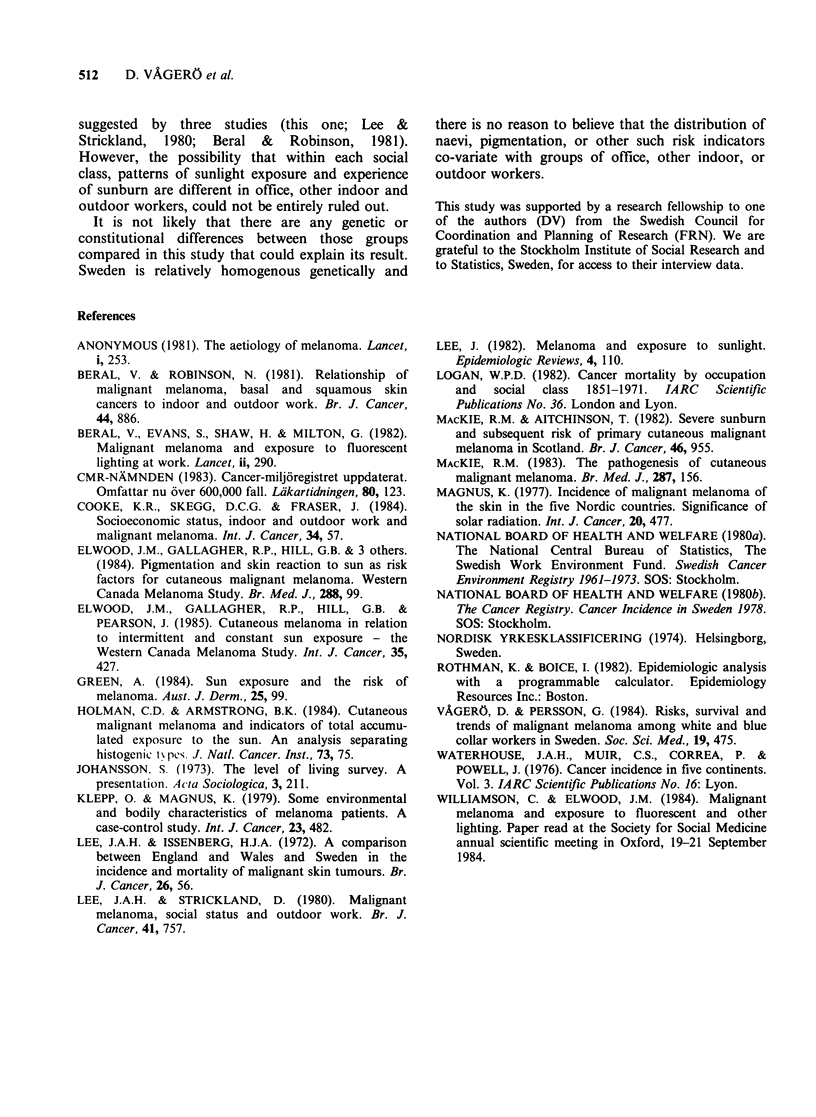


## References

[OCR_00541] Beral V., Evans S., Shaw H., Milton G. (1982). Malignant melanoma and exposure to fluorescent lighting at work.. Lancet.

[OCR_00535] Beral V., Robinson N. (1981). The relationship of malignant melanoma, basal and squamous skin cancers to indoor and outdoor work.. Br J Cancer.

[OCR_00549] Cooke K. R., Skegg D. C., Fraser J. (1984). Socio-economic status, indoor and outdoor work, and malignant melanoma.. Int J Cancer.

[OCR_00560] Elwood J. M., Gallagher R. P., Hill G. B., Pearson J. C. (1985). Cutaneous melanoma in relation to intermittent and constant sun exposure--the Western Canada Melanoma Study.. Int J Cancer.

[OCR_00567] Green A. (1984). Sun exposure and the risk of melanoma.. Australas J Dermatol.

[OCR_00571] Holman C. D., Armstrong B. K. (1984). Cutaneous malignant melanoma and indicators of total accumulated exposure to the sun: an analysis separating histogenetic types.. J Natl Cancer Inst.

[OCR_00581] Klepp O., Magnus K. (1979). Some environmental and bodily characteristics of melanoma patients. A case-control study.. Int J Cancer.

[OCR_00597] Lee J. A. (1982). Melanoma and exposure to sunlight.. Epidemiol Rev.

[OCR_00592] Lee J. A., Strickland D. (1980). Malignant melanoma: social status and outdoor work.. Br J Cancer.

[OCR_00606] MacKie R. M., Aitchison T. (1982). Severe sunburn and subsequent risk of primary cutaneous malignant melanoma in scotland.. Br J Cancer.

[OCR_00615] Magnus K. (1977). Incidence of malignant melanoma of the skin in the five Nordic countries: significance of solar radiation.. Int J Cancer.

[OCR_00640] Vågerö D., Persson G. (1984). Risks, survival and trends of malignant melanoma among white and blue collar workers in Sweden.. Soc Sci Med.

